# Expression of PAWR predicts prognosis of ovarian cancer

**DOI:** 10.1186/s12935-020-01704-y

**Published:** 2020-12-14

**Authors:** Jiahong Tan, Kangjia Tao, Xu Zheng, Dan Liu, Ding Ma, Qinglei Gao

**Affiliations:** 1 Cancer Biology Research Center (Key Laboratory of the Ministry of Education), Tongji Hospital, Tongji Medical College, Huazhong University of Science and Technology, 1095 Jiefang Ave, Wuhan, 430030 People’s Republic of China; 2 Department of Obstetrics and Gynecology, Tongji Hospital, Tongji Medical College, Huazhong University of Science and Technology, Wuhan, 430030 People’s Republic of China

**Keywords:** Drug responsiveness, Ovarian cancer, PAWR, Survival

## Abstract

**Background:**

Ovarian cancer greatly threatens the general health of women worldwide. Implementation of predictive prognostic biomarkers aids in ovarian cancer management.

**Methods:**

Using online databases, the general expression profile, target-disease associations, and interaction network of PAWR were explored. To identify the role of PAWR in ovarian cancer, gene correlation analysis, survival analysis, and combined analysis of drug responsiveness and PAWR expression were performed. The predictive prognostic value of PAWR was further validated in clinical samples.

**Results:**

PAWR was widely expressed in normal and cancer tissues, with decreased expression in ovarian cancer tissues compared with normal tissues. PAWR was associated with various cancers including ovarian cancer. PAWR formed a regulatory network with a group of proteins and correlated with several genes, which were both implicated in ovarian cancer and drug responsiveness. High PAWR expression denoted better survival in ovarian cancer patients (OS: HR = 0.84, P = 0.0077; PFS, HR = 0.86, P = 0.049). Expression of PAWR could predict platinum responsiveness in ovarian cancer and there was a positive correlation between PAWR gene effect and paclitaxel sensitivity. In 12 paired clinical samples, the cancerous tissues exhibited significantly lower PAWR expression than matched normal fallopian tubes. The predictive prognostic value of PAWR was maintained in a cohort of 50 ovarian cancer patients.

**Conclusions:**

High PAWR expression indicated better survival and higher drug responsiveness in ovarian cancer patients. PAWR could be exploited as a predictive prognostic biomarker in ovarian cancer.

## Background

Ovarian cancer ranks seventh the most common cancer, causing 152,000 deaths annually worldwide (4.3% of all cancer deaths), and the 5-year survival rate for ovarian cancer has remained unchanged for decades [1]. High-grade serous ovarian cancer is the most common histological subtype, which is thought to originate from the fallopian tubes and is characterized by nearly universal TP53 gene abnormalities [1]. The introductions of platinum in 1976 and paclitaxel in 1993 have greatly improved the outcomes of ovarian cancer patients [1–3]. Currently, combination regimens containing carboplatin and paclitaxel are the global standard of care [4]. A response rate of approximately 80% has been noted in initial chemotherapy [1]. However, the majority of patients develop resistance and recurrence [1, 5]. Implementation of predictive prognostic biomarkers could facilitate the management of ovarian cancer [1, 4–7].

PRKC apoptosis WTI regulator (PAWR), also known as Par-4, is a leucine zipper domain protein implicated in various cancers, including prostate cancer, bladder cancer, breast cancer, endometrial cancer, and leukemia [8–13]. PAWR is localized in the cytoplasm of diverse normal tissue cells and in both the cytoplasm and the nucleus of many tumor cells [10]. Endogenous PAWR is essential for apoptosis induction in response to a variety of exogenous insults [14]. Besides, as a tumor suppressor, PAWR induces apoptosis in a cancer-specific manner [9, 15]. PAWR is necessary for PTEN-inducible apoptosis, and inhibition of the PI3K/AKT signaling leads to PAWR-dependent apoptosis [16]. The PTEN/PI3K/AKT pathway is altered in more than 55% of ovarian cancer cases, which also affects the drug responsiveness of ovarian cancer [1, 17]. Moreover, the chemo-sensitivity and response to cisplatin and docetaxel of some cancers involve PAWR [14, 18, 19]. Therefore, PAWR may provide a feasible biomarker to predict drug responsiveness of ovarian cancer and a cancer-selective target for therapeutics.

In this study, we profiled PAWR expression in normal and tumor tissues of the human body and explored the target-disease associations of PAWR with various cancers. The protein regulatory network and gene correlations of PAWR were further analyzed to construct the regulatory network of PAWR. The prognostic effect for survival and the predictive value for drug responsiveness of PAWR were also assessed to illustrate its function in ovarian cancer.

## Methods

### HPA analysis

The HPA database (https://www.proteinatlas.org), which focuses on different aspects of the genome-wide analysis of human proteins, comprehensively maps all the human proteins in cells, tissues, and organs [20]. The global expression patterns of PAWR in all major tissues and organs in the human body were profiled using HPA.

### GEPIA analysis

The GEPIA database (http://gepia.cancer-pku.cn) is an interactive web server for analyzing the RNA-sequencing expression data of 9736 tumor samples and 8587 normal samples [21]. Comparison of PAWR expression between TCGA and GTEx samples in different cancer subtypes was performed using GEPIA. Besides, the correlation between PAWR and other genes was assessed in GEPIA and Pearson’s coefficient was calculated.

### c-BioPortal analysis

c-BioPortal (https://www.cbioportal.org) is an online database for interactive exploration of multidimensional cancer genomic datasets [22]. By using c-BioPortal, we obtained data on the mutations, copy number alterations, and mRNA expression of PAWR. The mutation spectrum and clinicopathological characteristics of ovarian cancer patients included in the TCGA dataset were also retrieved from c-BioPortal.

### Evaluation of target-disease associations

To identify and visualize potential drug targets associated with diseases, the Open Targets platform (https://www.targetvalidation.org) integrates evidence from genetics, genomics, transcriptomics, drugs, animal models, and scientific literatures [23]. Using the Open Targets platform, we scored and ranked the target-disease associations of PAWR.

### Gene Ontology (GO) analysis

The GO resource (http://geneontology.org) provides up-to-date and comprehensive knowledge concerning the functions of genes from many different organisms, from *Homo sapiens* to bacteria, facilitating biological research [24]. GO terms involving PAWR were searched in the GO database.

### Construction of protein–protein interaction network

The STRING database (https://string-db.org) collects, scores, and integrates all publicly available sources of protein–protein interaction information and complements these with computational predictions [25]. The latest version currently covers 24,584,628 proteins from 5090 organisms. The protein–protein interaction network of PAWR was constructed using STRING.

### Survival analysis

The Kaplan–Meier plotter database (KM plotter) (https://kmplot.com/analysis/), which is capable of assessing the effects of 54,000 genes on survival in 21 cancer types, integrates gene expression and clinical data simultaneously [26]. To determine the prognostic value of PAWR, KM plotter was analyzed, and hazard ratio with 95% confidence interval and log-rank P value were calculated.

### Oncomine analysis

Oncomine (https://www.oncomine.org/resource/main.html) is an integrated gene chip and data mining platform, which contains more than 90,000 cancer samples and 12,000 normal samples [27]. We used Oncomine to examine the expression levels of PAWR related to different clinical outcomes and recurrence statuses. PAWR expression under different paclitaxel responsiveness was also analyzed by using Oncomine.

### UALCAN analysis

UALCAN (http://ualcan.path.uab.edu) is an interactive web portal, which allows in-depth analyses of TCGA gene expression data. [28]. Using UALCAN, we analyzed the relative expression of PAWR across various tumor subgroups based on individual cancer stages. The differences among different tumor stages were evaluated using one-way ANOVA.

### Cancer dependency analysis

The Dependency Map portal (DepMap) (https://depmap.org/portal/) systematically identifies genetic dependencies of cancers and small molecule sensitivities and discovers biomarkers that predict them [29]. By exploring DepMap, we predicted the correlation between PAWR gene effect and drug sensitivity of ovarian cancer cell lines. Pearson’s correlation test was performed to evaluate the statistical significance.

### Predictive value analysis

The ROC plotter (http://www.rocplot.org) links gene expression and response to therapy using transcriptome-level data of 3104 breast cancer patients and 2369 ovarian cancer patients, enabling efficient validation of predictive biomarkers [30]. Treatment response was identified based on relapse-free survival at 12 months. PAWR expression was compared between platinum responders and non-responders using Mann–Whitney test. A receiver operating characteristic curve was drawn to analyze the predictive value of PAWR for platinum responsiveness. The area under the curve (AUC) and P value were also calculated.

### Immunohistochemistry

Formalin-fixed, paraffin-embedded ovarian cancer tissues were subjected to immunohistochemical analysis to determine the expression of PAWR. Briefly, an Avidin–Biotin Complex Vecastatin Kit (SP-9001, Zsgb-Bio, China) was used according to the manufacturer’s instructions. After deparaffinization and rehydration in a series of xylene and graded ethanol solutions, heat-induced antigen retrieval was performed in Tris–EDTA buffer (pH 9.0, G1203, Servicebio, China). Tumor slides were incubated with a primary antibody of PAWR (1:100 20688-1-AP, Proteintech, China) at 4 ℃ overnight. Following diaminobenzidine (G1212-200T, Servicebio) detection, hematoxylin was counterstained. To quantify PAWR expression, an immunoreactivity scoring system (HSCORE, range from 0 to 3) was used as described previously [31]. Concisely, *i* denotes the staining intensity of tumor cells (0, absent; 1, weak; 2, moderate; and 3, strong), and *Pi* represents the percentage of cells at the corresponding intensity, HSCORE = ∑*Pi* × *i*. All slides were scored by two investigators, who were blinded to all clinicopathological variables. The median HSCORE was used as the expression cut-off to classify patients into subgroups for further analysis (HSCORE ≤ 1, low expression; HSCORE > 1, high expression).

### Clinical samples

All clinical samples were obtained with signed informed consent from the Gynecology Department of Tongji Hospital, Tong Medical College, Huazhong University of Science and Technology. The patients were diagnosed with high-grade serous ovarian cancer and underwent initial debulking surgery without preoperative chemotherapy or radiotherapy at the Gynecology Department of Tongji Hospital. This study was approved by the Ethical Committee of Tongji Medical College, Huazhong University of Science and Technology. A total of 50 patients with detailed therapeutic and follow-up information were included, of whom 12 had paired normal fallopian tubes. The follow-up period lasted for 80 months (from January 2014 until September 2020). All patients received first-line platinum-containing chemotherapy. Platinum resistance was defined as recurrence within 6 months of completion of first-line treatment, while platinum sensitivity was defined as recurrence after more than 6 months [5].

### Statistical analysis

Bioinformatic analyses of databases were described above. A two-sided Student’s *t*-test was used to compare differences between groups. Survival analysis was performed with Kaplan–Meier curves using log-rank test. Fisher’s exact test and Chi-squared test were used to evaluate whether PAWR expression was correlated with clinicopathological characteristics of ovarian cancer patients. Data were plotted and analyzed using GraphPad Prism 7 (GraphPad Software, CA) and presented as the mean ± SD. Significance was assessed at the level of P < 0.05 and denoted as follows: *P < 0.05, **P < 0.01, ***P < 0.001, and ****P < 0.0001.

## Results

### Expression profile of PAWR

**Fig. 1:**
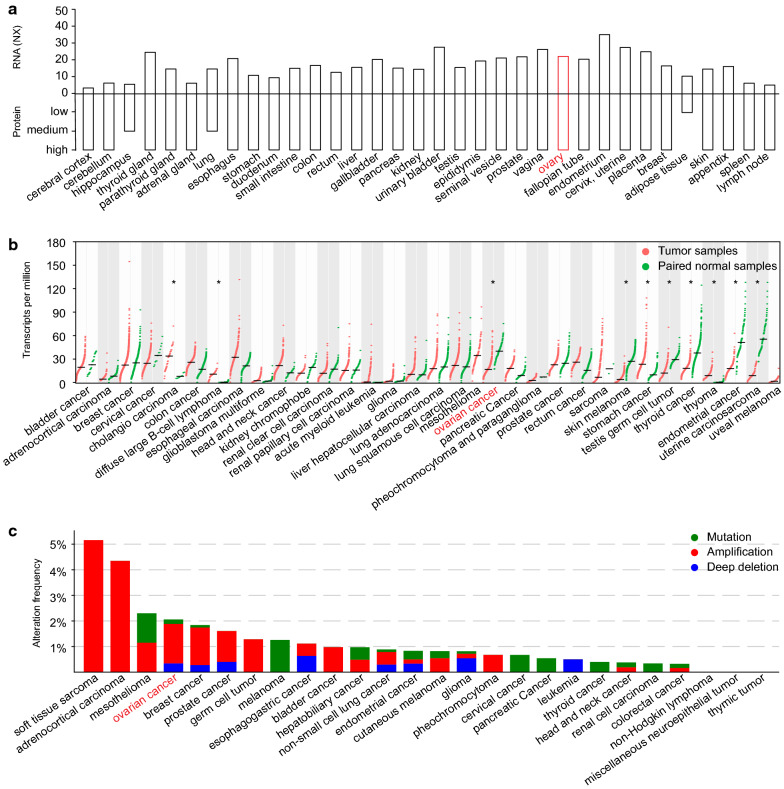
Expression profile of PAWR. **a** HPA analysis depicting mRNA and protein expression patterns of PAWR in 34 tissues (NX, normalized expression). **b** Comparison of PAWR expression in TCGA tumor samples and matched GTEx normal samples in GEPIA (one-way ANOVA). **c** Alteration frequency analysis of PAWR in TCGA cancers in c-BioPortal. P value was denoted as * P < 0.05, ** P < 0.01, *** P < 0.001, and **** P < 0.0001

To gain general insight concerning PAWR, we searched the HPA database and obtained the mRNA and protein expression information of PAWR in 34 tissues and organs in the human body (Fig. 1a). The Consensus RNA data integrated three different data sources, namely, HPA, GTEx, and FANTOM5, producing a normalized expression (NX). Normal ovary had a high PAWR protein expression and an NX of 22.1. Then, we compared PAWR expression in TCGA tumor samples and matched GTEx normal samples using GEPIA (Fig. 1b). Among the 33 tumor subtypes archived in GEPIA, 10 kinds had statistically significant expression differences between tumor and normal tissues, including ovarian cancer. The alteration frequency of PAWR was further profiled in TCGA cancers using c-BioPortal (Fig. 1c). In the TCGA dataset, nearly 2% of ovarian cancer patients had PAWR alterations. Among these patients, more than half of them had PAWR amplification, followed by PAWR deep deletion and then PAWR mutation. We also explored the mutation profile of PAWR in a collection of TCGA cancers (Additional file 1: Figure S1a) and in individual cancer types (Additional file 1: Figure S1b). Overall, PAWR is widely expressed in normal and cancer tissues.

### PAWR associates with various cancers

**Fig. 2:**
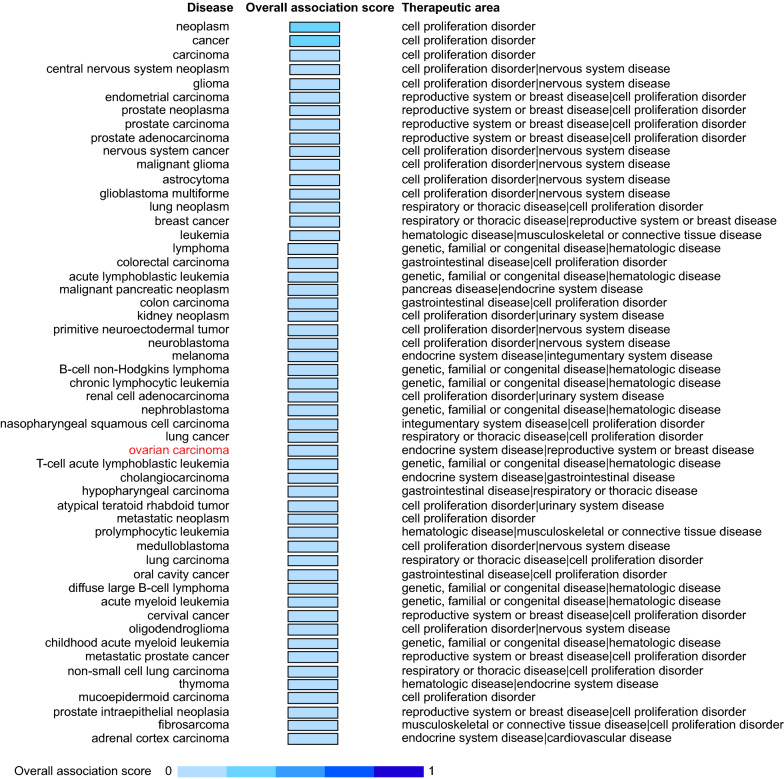
Target-disease associations of PAWR and various cancers. By analyzing the Open Targets platform, we found that 53 kinds of cancerous diseases were associated with PAWR. After integrating evidence from genetics, genomics, transcriptomics, drugs, animal models, and literatures, every target-disease association was scored and ranked

Identification of target-disease associations assists with drug discovery [23]. Information concerning PAWR, including genetic associations, somatic mutations, drugs, pathways & systems biology, RNA expression, text mining, and animal models, was integrated, scored, and ranked in the Open Targets platform. Then, an overall association score was assigned for every specific target-disease association. A total of 123 kinds of diseases were associated with PAWR, among which 53 (43.09%) were cancerous (Fig. 2). Ovarian carcinoma, which is included in the therapeutic areas of endocrine system disease, reproductive system or breast disease, cell proliferation disorder, and urinary system disease, appeared in the rank list. GO analysis further revealed the gene function of PAWR (Table 1). There were five GO terms involving PAWR, some of which are generally implicated in cancer, such as negative regulation of transcription by RNA polymerase II.

**Table 1:** GO terms involving PAWR

Gene/product	Gene/product name	GO class	Contributor	Organism
PAWR	PRKC apoptosis WT1 regulator protein	negative regulation of transcription by RNA polymerase II	Ensembl	Homo sapiens
PAWR	PRKC apoptosis WT1 regulator protein	nuclear chromatin	Ensembl	Homo sapiens
PAWR	PRKC apoptosis WT1 regulator protein	transcription corepressor activity	PINC	Homo sapiens
PAWR	PRKC apoptosis WT1 regulator protein	actin binding	UniProt	Homo sapiens
PAWR	PRKC apoptosis WT1 regulator protein	protein binding	UniProt	Homo sapiens

*GO* gene ontology

### Regulatory network of PAWR

**Fig. 3:**
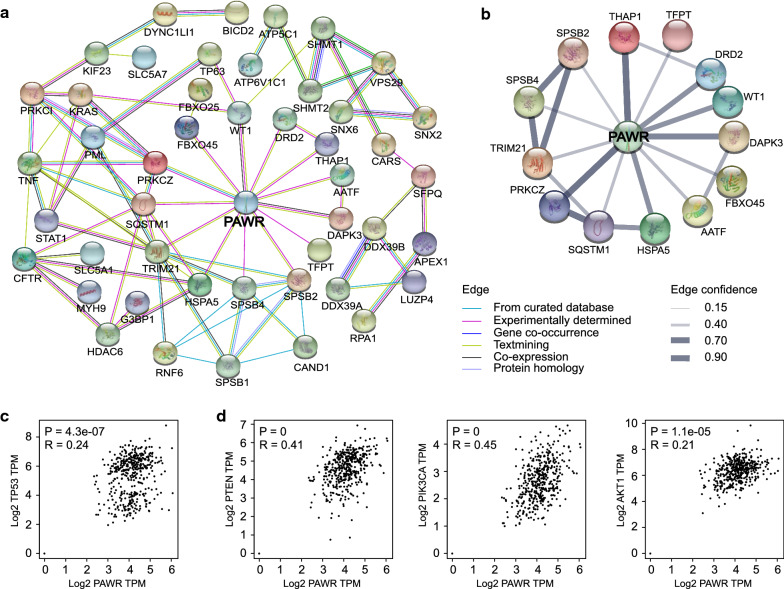
Protein interactions and gene correlations of PAWR. **a** The protein–protein interaction network of PAWR was constructed using STRING. **b** The interaction confidences of 13 proteins that directly interacted with PAWR were calculated and visualized. **c** Correlation analysis of PAWR and TP53 was performed in the TCGA dataset using GEPIA. (Pearson’s correlation test; TPM, transcripts per million). **d** Correlations between PAWR and the three hub genes of the PTEN/PI3K/AKT signaling axis were analyzed using GEPIA. (Pearson’s correlation test; TPM, transcripts per million)

By exploring the Gene Resource of the National Center for Biotechnology Information, we identified 80 proteins that might interact with PAWR (Additional file 2: Table S1). Subsequently, their gene symbols were imported into STRING for visualization (Fig. 3a). A total of 13 proteins directly interacted with PAWR. For these 13 proteins, confidence scores were calculated (Fig. 3b). The interaction confidence for THAP1, PRKCZ, and DAPK3 ranked top three. We then explored the gene correlations of PAWR in ovarian cancer. TP53, an important tumor suppressor gene in ovarian cancer [1], had a significantly positive correlation with PAWR (R = 0.24, P = 4.3e−07) (Fig. 3c). The PTEN/PI3K/AKT signaling pathway, implicated in cell proliferation, apoptosis, metastasis, and chemo-resistance of ovarian cancer, has been reported to correlate with PAWR [4, 15, 32]. Using GEPIA, we found positive correlations between PAWR and the three hub genes of this axis (Fig. 3d).

### High PAWR expression denotes better survival in ovarian cancer

**Fig. 4:**
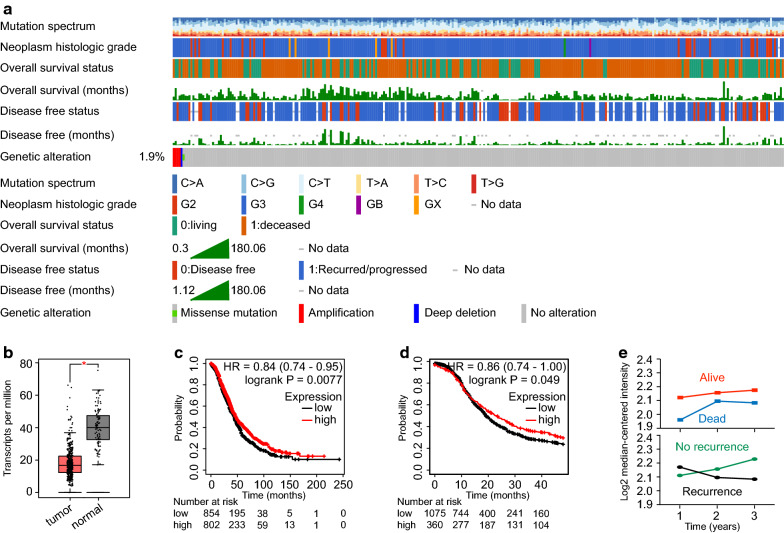
High PAWR expression correlates with better survival in ovarian cancer. **a** Clinical characteristics of ovarian cancer patients and genetic signatures of PAWR were studied in detail in the TCGA dataset using c-BioPortal. **b** Expression of PAWR in ovarian cancer tissues and paired normal tissues was compared using GEPIA (one-way ANOVA). Survival analysis was performed using KM plotter. Kaplan–Meier survival curves for OS (**c**) and PFS (**d**) were shown (log-rank test). **e** PAWR expression under different survival outcomes and recurrence statuses was explored in Oncomine. P value was denoted as *P < 0.05, **P < 0.01, ***P < 0.001, and ****P < 0.0001

To clarify the definite role of PAWR in ovarian cancer, clinical characteristics of ovarian cancer patients and genetic signatures of PAWR were studied in detail in the TCGA dataset and depicted in onco-prints (Fig. 4a). The detailed mutation profile of PAWR in ovarian cancer was also studied in c-BioPortal (Additional file 3: Figure S2). According to GEPIA, ovarian cancer samples expressed significantly lower PAWR than paired normal samples (Fig. 4b). By survival analysis, high PAWR expression indicated better overall survival (OS) than low PAWR expression (HR = 0.84, P = 0.0077) (Fig. 4c). Consistently, high PAWR expression predicted longer progression-free survival (PFS) (HR = 0.86, P = 0.049) (Fig. 4d). Similar results were obtained in ovarian cancer patients receiving optimal debulking surgery, wherein patients with high PAWR expression exhibited longer OS (Additional file 4: Figure S3a) and PFS (Additional file 4: Figure S3b). By exploring UALCAN, we found that there was no significant difference in PAWR expression among different tumor stages of ovarian cancer (Additional file 5: Figure S4a). However, after optimal debulking surgery, stage I/II patients with high PAWR expression had more favorable OS (Additional file 5: Figure S4b) and PFS (Additional file 5: Figure S4c). A consistent OS advantage was observed in stage III/IV patients receiving optimal debulking surgery (Additional file 5: Figure S4d). Although the difference in PFS did not reach the predefined threshold of statistical significance in stage III/IV patients, those patients with high PAWR expression tended to have longer PFS after optimal debulking surgery (Additional file 5: Figure S4e). The expression of PAWR under different survival and recurrence statuses was also examined in Oncomine (Fig. 4e). Patients with higher PAWR expression tended to live longer and relapse later. In summary, high PAWR expression denotes better survival of ovarian cancer patients.

### PAWR expression indicates drug responsiveness of ovarian cancer

**Fig. 5:**
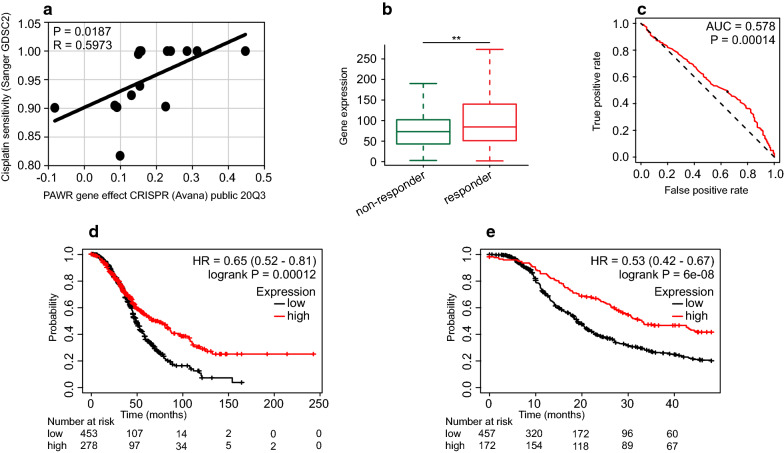
High PAWR expression indicates high platinum responsiveness in ovarian cancer. **a** The gene effect of PAWR on platinum responsiveness of ovarian cancer cell lines was analyzed using DepMap (Pearson’s correlation test). Platinum treatment response was identified based on relapse-free survival at 12 months in ROC plotter. **b** PAWR expression was compared between responders and non-responders (Mann–Whitney test). **c** A receiver operating characteristic curve was drawn to analyze the predictive value of PAWR for platinum responsiveness. Survival analysis was performed for ovarian cancer patients receiving platinum-containing chemotherapy after optimal debulking surgery. Kaplan–Meier survival curves for OS (**d**) and PFS (**e**) were depicted (log-rank test)

Since platinum agents constitute the standard of chemotherapy for ovarian cancer [1], we explored DepMap to identify the gene effect of PAWR on platinum responsiveness. There was a significant positive correlation between PAWR gene effect and cisplatin sensitivity of ovarian cancer cell lines (R = 0.5973, P = 0.0187) (Fig. 5a). In ROC plotter, we identified platinum treatment response based on relapse-free survival at 12 months. Platinum responders had higher PAWR expression than non-responders (Fig. 5b). Moreover, PAWR had a significant predictive value for platinum response (AUC = 0.578, P = 0.00014) (Fig. 5c). Paclitaxel is widely used in combination with platinum in treatment regimens for ovarian cancer [1]. In DepMap, PAWR gene effect was positively related to paclitaxel sensitivity of ovarian cancer cell lines (Additional file 6: Figure S5a). Consistent results were obtained when we performed an integrated analysis of six datasets in Oncomine, wherein paclitaxel-sensitive cell lines had a tendency toward elevated PAWR expression (Additional file 6: Figure S5b). In patients receiving platinum-containing chemotherapy after optimal debulking surgery, high PAWR expression predicted better OS (HR = 0.65, P = 0.00012) (Fig. 5d) and PFS (HR = 0.53, P = 6e−8) (Fig. 5e). A survival analysis in patients receiving paclitaxel-containing chemotherapy and optimal debulking surgery produced the same results (Additional file 6: Figure S5c, d). These results suggested that PAWR expression predicts platinum and paclitaxel responsiveness in ovarian cancer.

### Expression of PAWR predicts prognosis of ovarian cancer

**Fig. 6:**
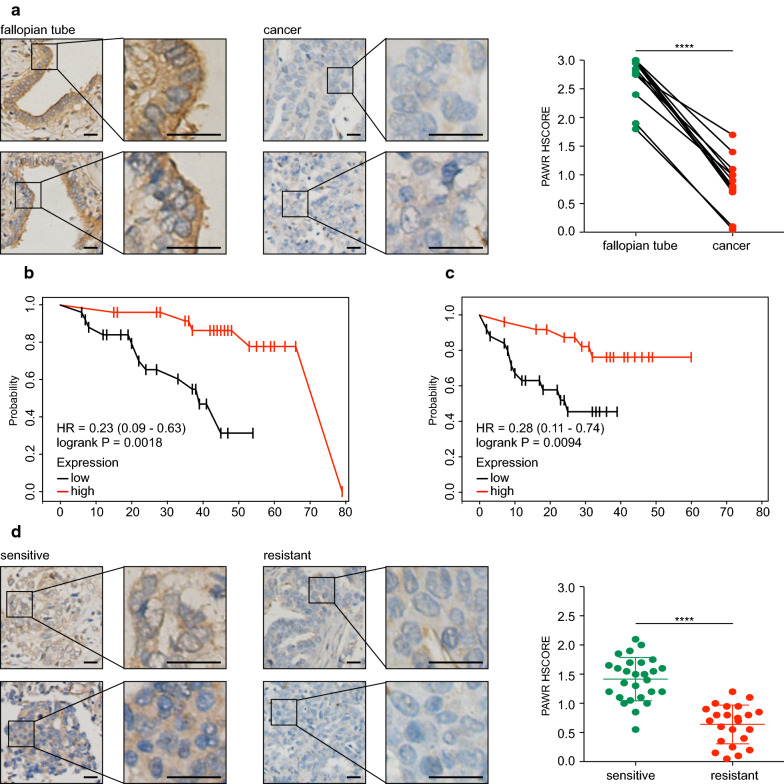
Expression of PAWR predicts prognosis of ovarian cancer. **a** Immunohistochemical staining for PAWR in 12 ovarian cancer tissues and paired normal fallopian tubes (Student’s *t*-test; bar, 20 μm). PAWR expression was also evaluated in another 38 clinical samples. The median HSCORE was used as the expression cut-off to classify patients into two subgroups (HSCORE ≤ 1, low expression; HSCORE > 1, high expression). Survival analysis was performed to compare these two groups, and survival curves were depicted for OS (**b**) and PFS (**c**) (log-rank test). According to the recurrence status within 6 months of completion of first-line treatment, patients were divided into two subgroups (recurrence within 6 months, resistant; no recurrence within 6 months, sensitive). **d** PAWR expression was compared between the sensitive group and the resistant group. (Student’s *t*-test; bar, 20 μm). P value was denoted as *P < 0.05, **P < 0.01, ***P < 0.001, and ****P < 0.0001

To further confirm its predictive value, PAWR expression was detected in clinical samples. 12 ovarian cancer tissues and paired normal fallopian tubes were subjected to immunohistochemical analysis (Fig. 6a). Consistent with the results obtained with GEPIA, ovarian cancer samples expressed significantly less PAWR than matched normal fallopian tubes. Another 38 tumor samples were also stained for PAWR. The median HSCORE was defined as the expression cut-off to classify patients into two subgroups for further analysis (HSCORE ≤ 1, low expression; HSCORE > 1, high expression). Patient characteristics were summarized and compared between these two groups (Table 2). Concordant with our earlier results, high PAWR expression predicted longer OS (HR = 0.23, P = 0.0018) (Fig. 6b) and PFS (HR = 0.28, P = 0.0094) (Fig. 6c). According to the recurrence status within 6 months of completion of first-line treatment, patients were divided into two groups (recurrence within 6 months, resistant; no recurrence within 6 months, sensitive). The platinum-sensitive group expressed significantly higher PAWR than the platinum-resistant group. To summarize, PAWR expression predicts prognosis of ovarian cancer.

**Table 2:** Characteristics of patients included in immunohistochemical analysis

Characteristics	total patients	PAWR high		PAWR low		*P* values
	(N = 50)	(N = 25)		(N = 25)		
	No	No	%	No	%	
Age at diagnosis						
≤ 50 years	12	5	20.00	7	28.00	0.7416^†^
> 50 years	38	20	80.00	18	72.00	
FIGO stage						
I	1	1	4.00	0	0.00	0.2695^‡^
II	10	5	20.00	5	20.00	
III	32	17	68.00	15	60.00	
IV	7	2	8.00	5	20.00	
Ascites						
Yes	25	14	56.00	11	44.00	0.5721^†^
No	25	11	44.00	14	56.00	

*FIGO* International Federation of Gynecology and Obstetrics

^†^Fisher’s exact test

^‡^Chi-squared test for trend

## Discussion

PAWR is a pro-apoptotic protein that has roles in both the intrinsic and extrinsic apoptotic pathways [14]. Here, we found that PAWR was expressed ubiquitously in normal and cancer tissues. Besides, PAWR was associated with various cancers, including ovarian cancer. PAWR formed an interactive regulatory network with a group of proteins and correlated with several genes, which were both implicated in ovarian cancer and drug responsiveness. Ovarian cancer tissues expressed significantly less PAWR than paired normal tissues. In ovarian cancer, high PAWR expression denoted better OS and PFS. Furthermore, PAWR expression predicted platinum and paclitaxel responsiveness in ovarian cancer. PAWR could be exploited as a predictive prognostic biomarker in ovarian cancer.

Integrating information from several databases, we profiled PAWR expression in humans. Decreased PAWR expression in ovarian cancer tissues compared with matched normal tissues suggested a tumor suppressor role of PAWR. The general target-disease associations of PAWR with various cancers further supported its prognostic and therapeutic value. In the regulatory network of PAWR, a panel of proteins and genes was implicated in ovarian cancer and affected the therapeutic response of ovarian cancer. For example, THAP1 functions as a proapoptotic factor [33]. PRKCZ, a member of the protein kinase C family, is involved in various cellular processes, including proliferation, differentiation, and secretion [34]. DAPK3 induces morphological changes in apoptosis and plays a role in the induction of apoptosis [35]. The pivotal tumor suppressor gene TP53 was correlated with PAWR in TCGA ovarian cancers. Similarly, PAWR had significant correlations with PTEN, PI3K, and AKT1, all of which are involved in ovarian cancer. All these results indicated that PAWR might be capable of predicting prognosis and drug responsiveness of ovarian cancer.

High PAWR expression predicted better OS and PFS in ovarian cancer patients. In the past few years, with improved perioperative support and multidisciplinary surgical teamwork, there has been a trend toward greater surgical intent with respect to optimal debulking and even total debulking [4]. In patients receiving optimal debulking surgery, the promotive effect of PAWR was maintained. Moreover, high PAWR expression was correlated with prolonged survival independent of tumor stage. Patients with high PAWR expression tended to live longer and recur later than those with low expression. The survival-promoting effect of PAWR was validated in our inhouse cohort. Owing to the lack of data, we were unable to analyze PAWR expression and platinum responsiveness integrally in Oncomine. However, PAWR was found to correlate with and predict platinum response of ovarian cancer patients in three other databases. For paclitaxel, the receiver operating characteristic curve in ROC plotter did not show statistical significance. However, the positive correlation between PAWR gene effect and paclitaxel sensitivity of ovarian cancer cell lines, the tendency toward elevated PAWR expression in paclitaxel-sensitive cell lines, and the survival advantage of patients with high PAWR expression further argued in favor of its role in paclitaxel response. Moreover, platinum and paclitaxel are generally used in combination in clinical applications [1, 4]. In our validation cohort, which contained patients who received carboplatin and paclitaxel combination regimens, high PAWR expression denoted better survival and platinum sensitivity. Therefore, we proposed that PAWR could predict prognosis of ovarian cancer. The TCGA dataset, which we primarily analyzed in the present study, comprised cases of high-grade serous ovarian cancer, the most common ovarian cancer subtype. All the clinical samples included were high-grade serous ovarian cancer. The role of PAWR in other subtypes of ovarian cancer remains to be elucidated. Tumor size is an important determinant of survival in ovarian cancer patients [36, 37]. The discordant data sources in some medical archives restrained the uniform assessment of tumor size. Consequently, we were unable to explore the correlation between tumor size and PAWR expression. However, high PAWR expression denoted prolonged survival and increased drug responsiveness. Therefore, it is reasonable to believe PAWR could be a feasible predictive prognostic biomarker in ovarian cancer.

Upfront treatment of ovarian cancer largely depends on debulking surgery to eliminate residual disease and cytotoxic chemotherapy [1]. Chemo-sensitivity assays or genetic screening arrays that establish drug sensitivity have been studied, but require further confirmation [4]. Evaluating PAWR expression provided a feasible approach to predict survival and drug responsiveness of ovarian cancer patients by simply performing immunohistochemical staining, which is convenient and cost-effective. Consistent with result for some other tissues [13, 15], PAWR was expressed in the cytoplasm in cells of normal fallopian tubes, while there was both cytoplasmic and nuclear expression of PAWR in cancer samples. PAWR can also be secreted spontaneously by normal and cancer cells in culture [38], providing the possibility of detection by non-invasive liquid biopsy. However, the current sample size was small. Before clinical implementation, large preclinical studies are needed. Another limitation is the lack of specific drugs targeting PAWR. We will explore inhibitors of PI3K and AKT for PAWR-targeted therapy in future research.

## Conclusions

In summary, PAWR is widely expressed in normal and cancer tissues and is associated with various cancerous diseases. Ovarian cancer tissues express significantly less PAWR than matched normal tissues. High PAWR expression indicates better survival and higher drug responsiveness in ovarian cancer patients. PAWR could be exploited as a predictive prognostic biomarker in ovarian cancer, which warrants further investigation.

## Supplementary Information

**Additional file 1: Figure S1.** Mutation profile of PAWR in various cancers. (a) Integrated mutation profile of PAWR in different TCGA cancers. (b) Mutation profile of PAWR in individual cancer types.

**Additional file 2: Table S1.** Proteins having potential interactions with PAWR.

**Additional file 3: Figure S2.** Mutation profile of PAWR in ovarian cancer. The detailed mutation profile of PAWR in TCGA ovarian cancers was studied using c-BioPortal.

**Additional file 4: Figure S3.** PAWR expression predicts survival of ovarian cancer patients after optimal debulking surgery. Ovarian cancer patients undergoing optimal debulking surgery were classified into two subgroups based on PAWR expression. Survival analysis was performed using KM plotter. Survival curves were depicted for OS (a) and PFS (b) (log-rank test).

**Additional file 5: Figure S4.** PAWR expression predicts survival of ovarian cancer patients regardless of tumor stage. (a) PAWR expression of the TCGA ovarian cancer patients was analyzed and compared among different tumor stages using UALCAN (one-way ANOVA). Survival analysis of stage I/II patients receiving optimal debulking surgery was performed. Kaplan–Meier survival curves for OS (b) and PFS (c) were shown (log-rank test). For stage III/IV patients undergoing optimal debulking surgery, survival analysis was performed using KM plotter. Kaplan–Meier survival curves for OS (d) and PFS (e) were depicted (log-rank test).

**Additional file 6: Figure S5.** High PAWR expression suggests high paclitaxel responsiveness in ovarian cancer. (a) The correlation between PAWR gene effect and paclitaxel responsiveness of ovarian cancer cell lines was analyzed in DepMap (Pearson’s correlation test). (b) PAWR expression under different paclitaxel responsiveness was analyzed in six datasets in Oncomine. Survival analysis was performed for ovarian cancer patients receiving paclitaxel-containing chemotherapy after optimal debulking surgery. Kaplan–Meier survival curves for OS (c) and PFS (d) were depicted (log-rank test).

## Data Availability

Not applicable.

## Abbreviations

AUC: Area under the curve
DepMap: Dependency Map portal
GO: Gene ontology
KM plotter: Kaplan–Meier plotter
NX: Normalized expression
OS: Overall survival
PFS: Progression-free survival
