# Supplementary figures and images for: Expression of PAWR predicts prognosis of ovarian cancer
Source: Cancer Cell Int. 2020 Dec 14;20:598. doi: 10.1186/s12935-020-01704-y (PMC7737345; doi:10.1186/s12935-020-01704-y)

a

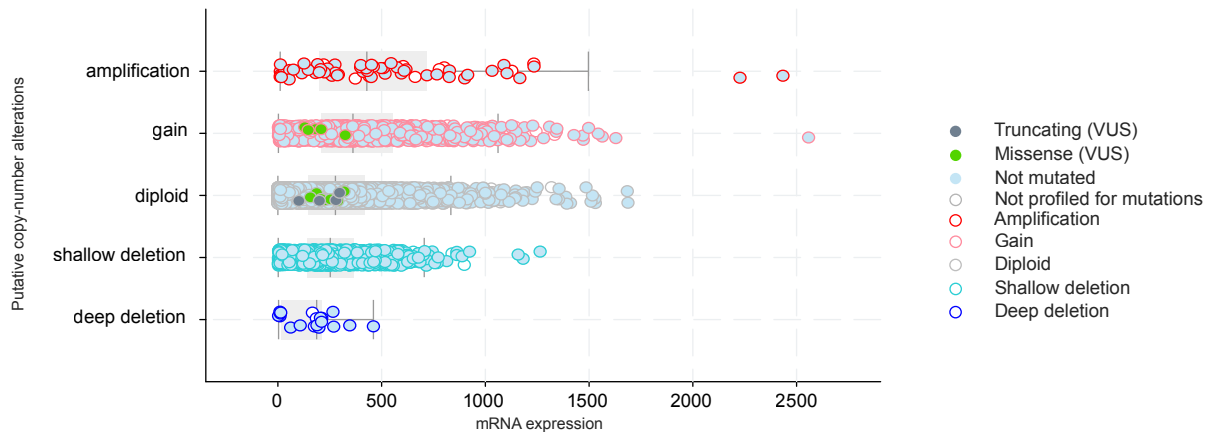

b

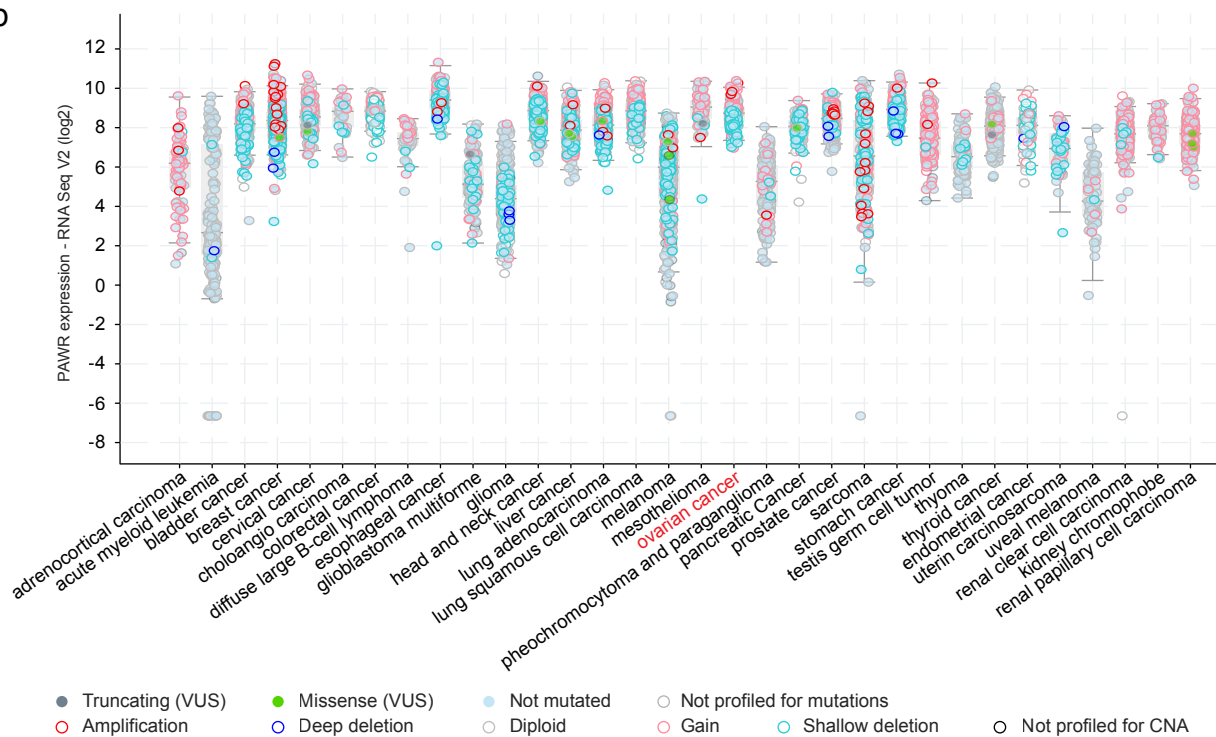

Supplement: Supplementary file 1 — Additional file 1: Figure S1. Mutation profile of PAWR in various cancers. (a) Integrated mutation profile of PAWR in different TCGA cancers. (b) Mutation profile of PAWR in individual cancer types. [file 12935_2020_1704_MOESM1_ESM.pdf]

Putative copy-number alterations

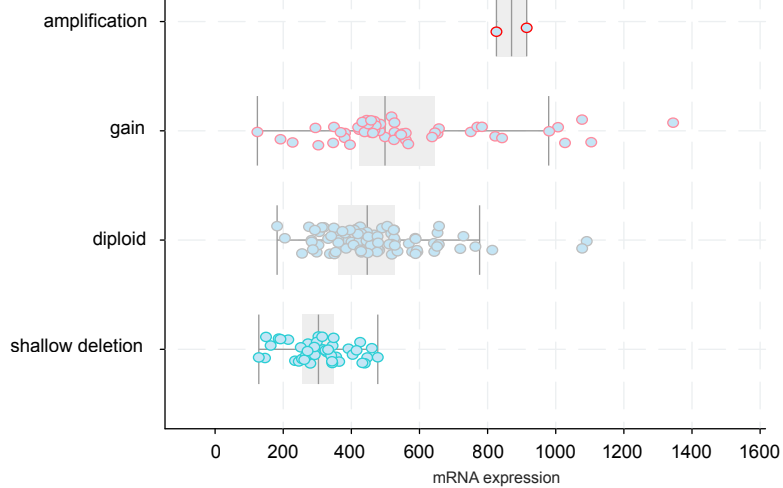

- Not mutated
- Amplification
- Gain
- Diploid
- Shallow deletion

Supplement: Supplementary file 3 — Additional file 3: Figure S2. Mutation profile of PAWR in ovarian cancer. The detailed mutation profile of PAWR in TCGA ovarian cancers was studied using c-BioPortal. [file 12935_2020_1704_MOESM3_ESM.pdf]

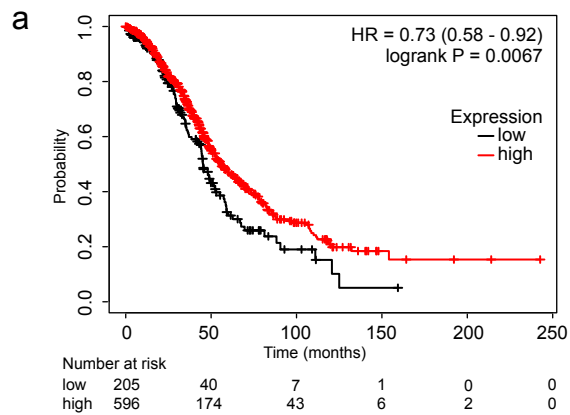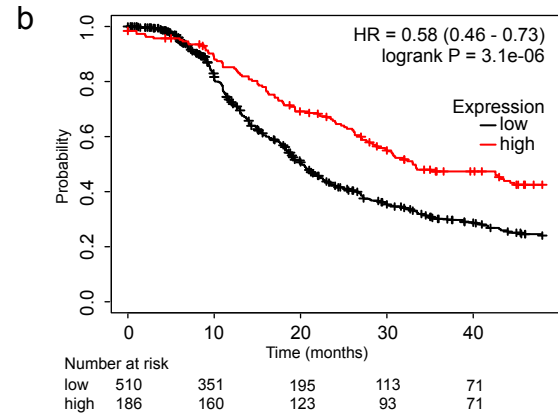

Supplement: Supplementary file 4 — Additional file 4: Figure S3. PAWR expression predicts survival of ovarian cancer patients after optimal debulking surgery. Ovarian cancer patients undergoing optimal debulking surgery were classified into two subgroups based on PAWR expression. Survival analysis was performed using KM plotter. Survival curves were depicted for OS (a) and PFS (b) (log-rank test). [file 12935_2020_1704_MOESM4_ESM.pdf]

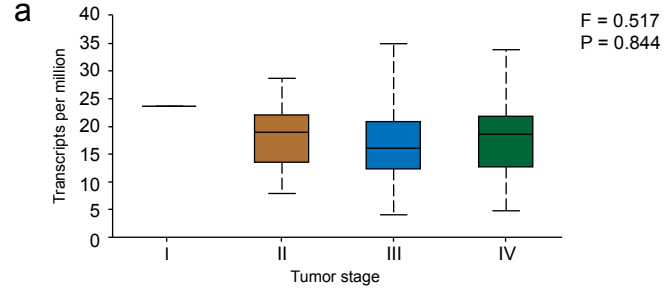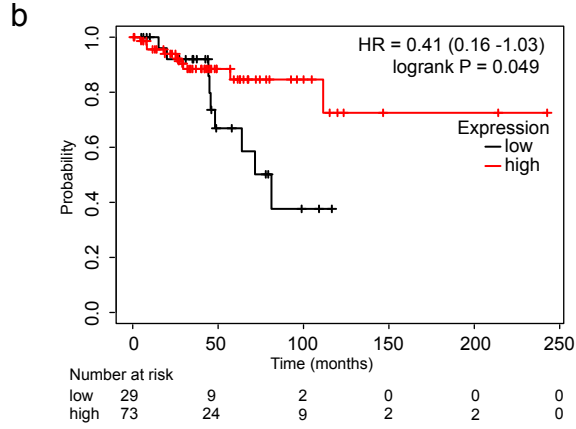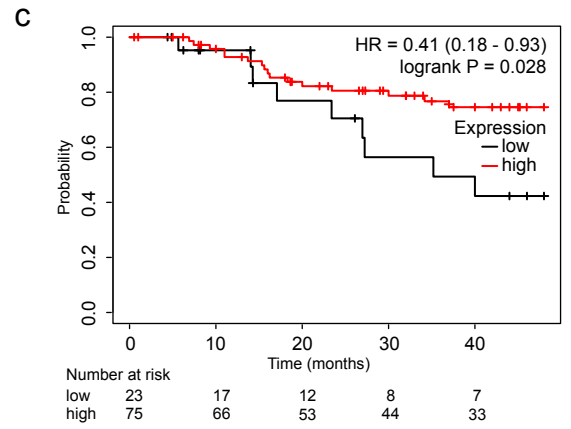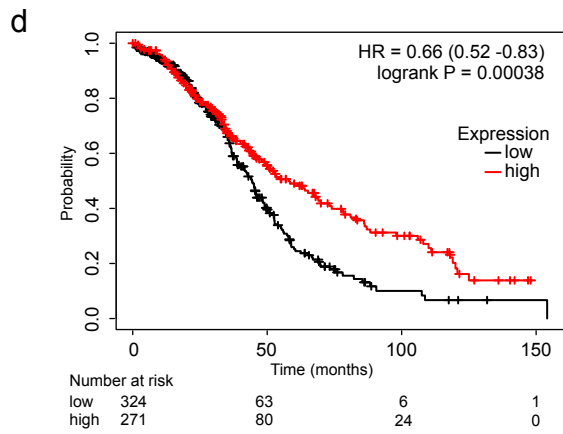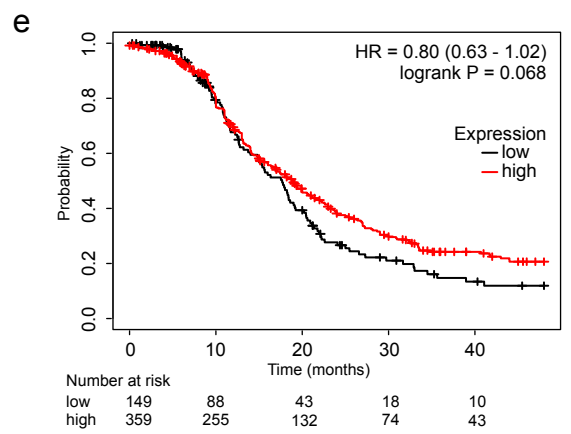

Supplement: Supplementary file 5 — Additional file 5: Figure S4. PAWR expression predicts survival of ovarian cancer patients regardless of tumor stage. (a) PAWR expression of the TCGA ovarian cancer patients was analyzed and compared among different tumor stages using UALCAN (one-way ANOVA). Survival analysis of stage I/II patients receiving optimal debulking surgery was performed. Kaplan–Meier survival curves for OS (b) and PFS (c) were shown (log-rank test). For stage III/IV patients undergoing optimal debulking surgery, survival analysis was performed using KM plotter. Kaplan–Meier survival curves for OS (d) and PFS (e) were depicted (log-rank test). [file 12935_2020_1704_MOESM5_ESM.pdf]

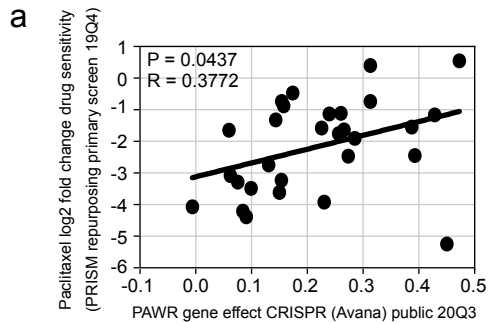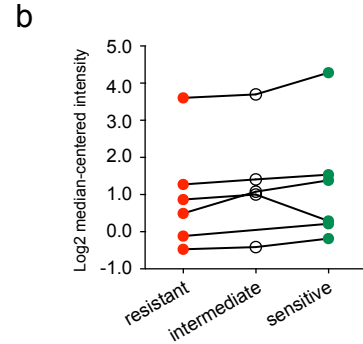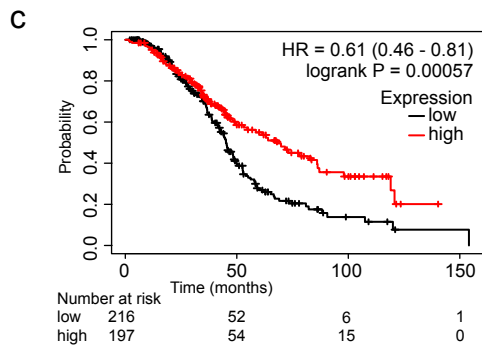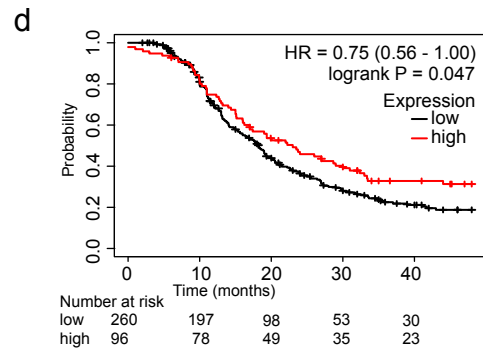

Supplement: Supplementary file 6 — Additional file 6: Figure S5. High PAWR expression suggests high paclitaxel responsiveness in ovarian cancer. (a) The correlation between PAWR gene effect and paclitaxel responsiveness of ovarian cancer cell lines was analyzed in DepMap (Pearson’s correlation test). (b) PAWR expression under different paclitaxel responsiveness was analyzed in six datasets in Oncomine. Survival analysis was performed for ovarian cancer patients receiving paclitaxel-containing chemotherapy after optimal debulking surgery. Kaplan–Meier survival curves for OS (c) and PFS (d) were depicted (log-rank test). [file 12935_2020_1704_MOESM6_ESM.pdf]
