# Supplementary material for: Expression of PAWR predicts prognosis of ovarian cancer
Source: Cancer Cell Int. 2020 Dec 14;20:598. doi: 10.1186/s12935-020-01704-y (PMC7737345; doi:10.1186/s12935-020-01704-y)
Supplement: Supplementary file 2 — Additional file 2: Table S1. Proteins having potential interactions with PAWR. [file 12935_2020_1704_MOESM2_ESM.docx]

| Name | Source | Description |
| --- | --- | --- |
| PAWR | BIND | Par-4 forms a homodimer. |
| PRKCI | BIND | Par-4 interacts with PKC-lambda/iota. This interaction was modelled on a demonstrated interaction between human Par-4 and Xenopus laevis PKC-lambda/iota. |
| PRKCZ | BIND | Par-4 interacts with PKC-zeta. This interaction was modelled on a demonstrated interaction between human Par-4 and rat PKC-zeta. |
| AATF | HPRD |  |
| ATP5F1C | HPRD |  |
| DAPK3 | HPRD |  |
| PAWR | HPRD |  |
| PML | HPRD |  |
| PRKCZ | HPRD |  |
| RRAS2 | HPRD |  |
| SLC5A7 | HPRD |  |
| TFPT | HPRD |  |
| THAP1 | HPRD |  |
| WT1 | HPRD |  |
| AATF | BioGRID | Affinity Capture-Western |
| APEH | BioGRID | Co-fractionation |
| APEX1 | BioGRID | Affinity Capture-RNA |
| ATP6V1C1 | BioGRID | Co-fractionation |
| BICD2 | BioGRID | Proximity Label-MS |
| CAND1 | BioGRID | Co-fractionation |
| CARS1 | BioGRID | Co-fractionation |
| CCDC8 | BioGRID | Affinity Capture-MS |
| CEP128 | BioGRID | Proximity Label-MS |
| CEP290 | BioGRID | Proximity Label-MS |
| CFTR | BioGRID | Affinity Capture-MS |
| CTDP1 | BioGRID | Affinity Capture-MS |
| CEP350 | BioGRID | Affinity Capture-MS |
| DAPK3 | BioGRID | Affinity Capture-Western; Biochemical Activity; Two-hybrid |
| DDX39A | BioGRID | Co-fractionation |
| DDX39B | BioGRID | Affinity Capture-MS |
| DRD2 | BioGRID | Reconstituted Complex; Two-hybrid |
| DYNC1LI1 | BioGRID | Proximity Label-MS |
| DYRK1A | BioGRID | Affinity Capture-MS |
| EED | BioGRID | Affinity Capture-MS |
| FBXO25 | BioGRID | Affinity Capture-MS |
| FBXO45 | BioGRID | Affinity Capture-MS; Affinity Capture-Western; Biochemical Activity; Reconstituted Complex |
| G3BP1 | BioGRID | Affinity Capture-MS |
| H3-3A | BioGRID | Co-fractionation |
| HDAC6 | BioGRID | Affinity Capture-MS |
| HMG20A | BioGRID | Affinity Capture-MS |
| HSPA5 | BioGRID | Affinity Capture-MS; Affinity Capture-Western |
| IDE | BioGRID | Co-fractionation |
| IKBIP | BioGRID | Affinity Capture-MS |
| JMJD6 | BioGRID | Co-fractionation |
| KRAS | BioGRID | Affinity Capture-MS |
| KIF23 | BioGRID | Affinity Capture-MS |
| LUZP4 | BioGRID | Affinity Capture-MS |
| MAB21L2 | BioGRID | Affinity Capture-MS |
| MYH9 | BioGRID | Affinity Capture-MS |
| NIBAN2 | BioGRID | Co-fractionation |
| PAN2 | BioGRID | Affinity Capture-MS |
| PLEKHA4 | BioGRID | Affinity Capture-MS |
| PPM1G | BioGRID | Co-fractionation |
| PRKCZ | BioGRID | Affinity Capture-Western; Reconstituted Complex; Two-hybrid |
| RNF6 | BioGRID | Affinity Capture-MS |
| RPA1 | BioGRID | Affinity Capture-MS |
| SCOC | BioGRID | Affinity Capture-MS |
| SFPQ | BioGRID | Affinity Capture-MS |
| SHMT1 | BioGRID | Co-fractionation |
| SHMT2 | BioGRID | Co-fractionation |
| SLC25A41 | BioGRID | Affinity Capture-MS |
| SLC5A1 | BioGRID | Affinity Capture-Western |
| SLC5A7 | BioGRID | Affinity Capture-Western |
| SNX2 | BioGRID | Co-fractionation |
| SNX6 | BioGRID | Co-fractionation |
| SPSB1 | BioGRID | Protein-peptide |
| SPSB2 | BioGRID | Protein-peptide |
| SPSB4 | BioGRID | Protein-peptide |
| SQSTM1 | BioGRID | Affinity Capture-Western; Reconstituted Complex |
| STAT1 | BioGRID | Co-fractionation |
| SPSB2 | BioGRID | Reconstituted Complex |
| SPSB4 | BioGRID | Reconstituted Complex |
| THAP1 | BioGRID | Co-localization; Reconstituted Complex; Two-hybrid |
| TNF | BioGRID | Affinity Capture-MS |
| TP63 | BioGRID | Positive Genetic |
| TRIM21 | BioGRID | Affinity Capture-MS; Affinity Capture-Western |
| VIRMA | BioGRID | Affinity Capture-RNA |
| VPS29 | BioGRID | Co-fractionation |
| WT1 | BioGRID | Affinity Capture-Western; Reconstituted Complex; Two-hybrid |
| vIRF-1 | BioGRID | Affinity Capture-MS |

**Table S1. Proteins having potential interactions with PAWR.**
